# O-48 - ESTIMATING THE BURDEN OF SHIGA TOXIN-PRODUCING ESCHERICHIA COLI (STEC) IN SCOTLAND – A DATA LINKAGE STUDY (2015-2023)

**DOI:** 10.1093/pubmed/fdag046.043

**Published:** 2026-07-09

**Authors:** Ms Susan Brownlie, Dr Alison Smith-Palmer, Dr Lesley Allison, Dr Anne Holmes, Dr Jane Horne, Dr Jacqui McElhiney

**Affiliations:** Public Health Scotland; Public Health Scotland; Scottish E. coli O157/STEC Reference Laboratory; Scottish E. coli O157/STEC Reference Laboratory; Food Standards Scotland; Food Standards Scotland

## Abstract

**Background:**

The incidence of Shiga toxin-producing Escherichia coli (STEC) in Scotland has been consistently high compared to other comparable countries, with the burden unevenly distributed across the population of Scotland.

**Objectives:**

To use data linkage to provide a detailed insight into the burden of infection of STEC in Scotland, including factors associated with severe clinical presentation, hospitalisation rates and duration, HUS and longer-term sequelae. The size of the dataset, together with the diverse linked data sources, provides a comprehensive understanding of the burden of STEC infection in Scotland and an evidence base to help inform future guidance and research. A similar data linkage study has already been completed for Campylobacter, and Salmonella linkage is currently underway, allowing comparisons to be drawn between these major gastrointestinal pathogens.

**Methods:**

A comprehensive data linkage study was undertaken using all laboratory confirmed cases of STEC in Scotland 2015-2023, totalling over 2500 cases with 43.5% requiring a hospital admission. Laboratory data, including Whole Genome Sequencing information on serotype, stx subtype and other key virulence genes, was linked to epidemiological, exposure and symptom data from surveillance questionnaires, hospitalisation data, cancer registry data, community prescribing data, Charlson comorbidity index, deprivation and rurality data.

**Results:**

Living in a rural area not only impacted the likelihood of contracting STEC but also influenced the strain contracted. Factors impacting severity of infection included predisposing conditions, key demographic data, as well as the particular strain of STEC, which was considered in the context of JEMRA classification of STEC infection.

**Conclusions:**

Rurality was an important factor, which has implications when considering risk communication for those living in and visiting rural communities and first line clinicians to whom they may present.

